# Cold Atmospheric Pressure Microplasma Pipette for Disinfection of Methicillin-Resistant *Staphylococcus aureus*

**DOI:** 10.3390/mi12091103

**Published:** 2021-09-14

**Authors:** Geunyoung Nam, Muhwan Kim, Yeonsook Jang, Sungbo Cho

**Affiliations:** 1 Department of Biomedical Engineering, Gachon Advanced Institute for Health Science & Technology, Gachon University, 191 Hambakmoe-ro, Incheon 21999, Korea; nanrmsdud@gachon.ac.kr; 2 Femto Science Inc., 557 Dongtangiheung-ro, Hwaseong-si 18469, Gyeonggi-do, Korea; mhkim@femtoscience.co.kr (M.K.); nancy@femtoscience.co.kr (Y.J.); 3 Department of Electronic Engineering, Gachon University, 1342 Seongnamdaero, Sujeong-gu, Seongnam-si 13120, Gyeonggi-do, Korea

**Keywords:** cold atmospheric pressure plasma, microplasma, antibacterial ability, disinfection, methicillin-resistant *Staphylococcus aureus*

## Abstract

Microbial infections should be controlled and prevented for successful wound healing and tissue regeneration. Various disinfection methods exist that use antibiotics, ultraviolet (UV), heat, radiation, or chemical disinfectants; however, cold atmospheric pressure plasma has exhibited a unique and effective antibacterial ability that is not affected by antibiotic resistance or pain. This study develops a cold atmospheric pressure microplasma pipette (CAPMP) that outputs an Ar plasma plume through a tube with an inner radius of 180 μm for disinfection in a small area. The CAPMP was evaluated using *Staphylococcus aureus* and methicillin-resistant *Staphylococcus aureus* diluted in liquid media, spread on solid agar, or covered by dressing gauze. An increase in the treatment time of CAPMP resulted in a decrease in the number of colonies of the grown microorganism (colony forming unit) and an increase in the disinfected area for both bacteria. The disinfection ability of CAPMP was observed when the bacteria were covered with dressing gauze and was dependent on the number of gauze layers.

## 1. Introduction

Atmospheric pressure, non-thermal plasma, or cold atmospheric pressure plasma (CAP), which generates plasma ions with unique physical and chemical characteristics, are increasingly being employed for diverse biophysical and medical applications [1,2,3,4,5]. A low-temperature plasma exhibits thermal non-equilibrium, and the temperature of its neutrals/ions is lower than that of its electrons; further, the plasma can be employed for the treatment of eukaryotic cells and tissues with the generated reactive species [6]. The characteristics and treatment effects of CAP are determined by the type and mixing ratio of the gases for discharge ignition, which leads to reactive oxygen species (ROS; O_2_^•–^, O_3_, H_2_O_2_, OH^•^, etc.), reactive nitrogen species (RNS; N_2_O_3_, NO^•^, NO_2_^•^, etc.), excited species, and UV radiation, etc. [7,8] ROS and RNS are known to cause irreversible damage to microbial growth and survival because they puncture and change the shape of the microbial cell membrane, resulting in the release of intracellular compounds, such as lipids, proteins, and DNA [9,10]. Recently, the application of CAP with a short treatment time of approximately 5 min was found to strongly inactivate microorganisms, satisfying certain requirements in the field of medicine, such as wound sterilization and dental care [11,12]. For better wound healing and therapeutic strategies, CAP has been applied to the surgical or necrotic sites of patients with chronic diseases to effectively reduce microbial communities and prevent infection [13,14,15,16].

The healing process of wounds is roughly categorized into three stages: inflammation, granulation tissue formation, and matrix formation and remodeling [17]. The success or failure of wound healing is determined at the inflammation stage, at which the tissues are vulnerable to infections with diverse pathogens [18]. Since microbial infections in the exposed dermis or tissues form a colony of microbes, slow down wound recovery, and lead to chronic wounds, microbial infections should be inhibited [18]. Thus, wound recovery is realized by the successful suppression of microbial infection [19,20]. The most frequently used method to suppress microbial infections is dressing the wound with sterile gauzes to protect the wound area from external contamination and to absorb exudate from the tissues [21,22]. To ensure that the dressing gauze effectively protects the wound tissues, new materials, including biopolymer, nanofibrous, or cellulose hydrogels, have been evaluated to enhance the biocompatibility and antibacterial effect of dressing gauzes [23,24,25]. However, micro-sized bacteria can penetrate through the dressing gauze and reach the wound area, and thus, bacterial infection and growth are still observed on the wound tissues [26,27]. Therefore, the wound should be kept clean to decrease the risk of contamination with microorganisms, and the dressing gauze should be regularly changed, which causes discomfort and occasionally pain [28].

Antibiotics can additionally be taken to prevent diseases caused by bacterial infections of the wound tissues. However, the use of antibiotics induces the evolution of microorganisms into antibiotic-resistant bacteria or superbugs, which are continually being discovered and are a threat to public health [29,30]. On the contrary, CAP successfully sterilizes methicillin-resistant *Staphylococcus aureus*, one of the representative antibiotic-resistant bacteria [31]. In an existing study, patients were treated with CAP for less than 5 min and experienced only slight discomfort and no pain [32]. The application of CAP is common for low-temperature sterilization and the surface modification of implantable devices, micro needles, wearable sensors, or nano processes [33,34,35,36]. Portable CAP has been commercialized for mobile wound care by the inactivation of multi-drug resistant organisms (Plasma care^®^, Terraplasma Medical GmbH, Munich, Germany), therapy for chronic diseases (including diabetic feet) (PlasmaDerm^®^, Plasma Technology for Health, Duderstadt, Germany), or superfine cleaning and treatment of the surface (PIEZOBRUSH^®^ PZ3, Relyon Plasma GmbH, Regensburg, Germany) [37,38,39].

This study develops a cold atmospheric pressure microplasma pipette (CAPMP) tool, called Plasma Pipette^®^, which has a long and narrow plasma plume, similar to that of plasma in a microwell plate or a tiny area of tissues [40]. The disinfection ability of CAPMP was evaluated using the following: 1. culture media with *Staphylococcus aureus* (*S. aureus*) and methicillin-resistant *Staphylococcus aureus* (MRSA) in the microwell; 2. agar plates were spread with *S. aureus* and MRSA; and 3. *S. aureus* and MRSA on the agar plates were covered with dressing gauze. The experimental results demonstrated the feasibility of Plasma Pipette^®^ to disinfect small areas, which is important for biomedical applications. The disinfection of the bacteria in the microwell was increased with an increase in the plasma treatment time. Further, the bacteria covered by the dressing gauze was disinfected after the plasma treatment.

## 2. Methods and Analysis

### 2.1. Plasma Pipette

**Figure 1:**
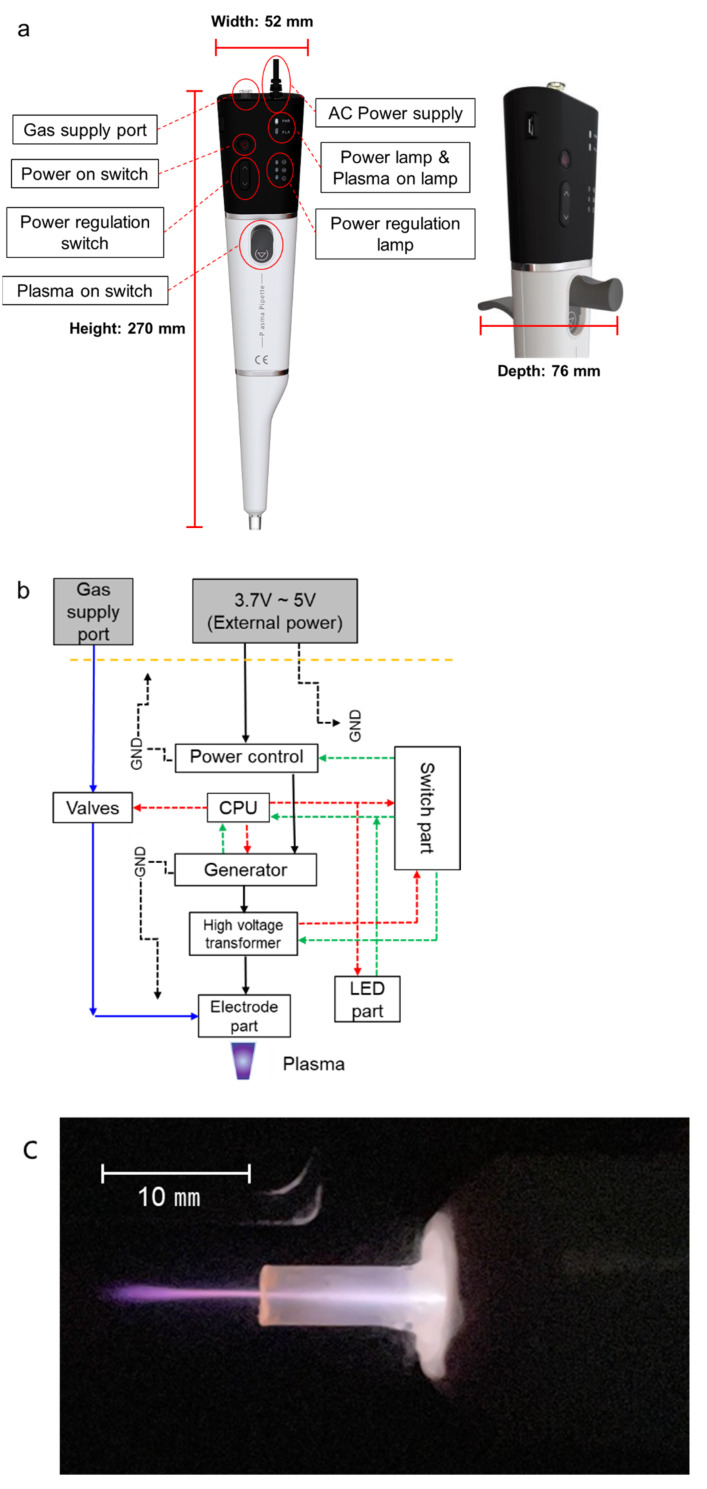
Components of Plasma Pipette^®^ and its schematic. (**a**) Each component of the pipette. (**b**) Block diagram of the internal structure of the pipette (blue line: gas supply path, black line: power line, red dot line: signal line, green dot line: feedback, and black dot–dash line: ground). (**c**) Pipette operation and plasma length.

CAPMP was developed using a microcontroller, a power regulator, a high-voltage transformer, an Ar gas supply port, and electrodes insulated by dielectrics [40]. Plasma Pipette^®^ weighs 375 g and has a pipette-shaped design (width: 52, depth: 76, and height: 270 mm) that facilitates plasma treatment on the micro area or microscopic components (Figure 1). The pipette can be powered by an adapter with a DC output of 5 V, 4 A, and an AC input of 100–240 V 50/60 Hz (1.2 A max.). The possible continuous operation time for a Plasma Pipette^®^ is more than 40 minutes. The voltage required to induce plasma discharge can be amplified to an average of 2.3 kV, with a frequency of 80 kHz, by a high-voltage transformer. When the plasma is generated, the Ar plasma plume is discharged through a tube with an inner radius of 180 μm, and the discharged plasma is output from the plasma pipette tip with an inner radius of 750 μm. The length of the plasma plume is over 10 mm, depending on the pressure of gas regulator connected to the gas supply port.

### 2.2. Bacteria Preparation

*S. aureus* and MRSA bacteria were incubated in sterile Luria-Bertani (LB) (Becton, Dickinson and Company, Franklin Lakes, NJ, USA) liquid medium for one day, and then transferred to LB agar plates. The transferred microorganisms were cultured at 37 °C to form colonies and stored in a refrigerator at 2 °C. The colonies were diluted in liquid LB media, and the concentration of cells in the suspension was estimated in terms of optical density (OD) at 595 nm using a spectrophotometer (EPOCH2, BioTek, Winooski, VT, USA) [41].

### 2.3. Plasma Treatment

**Figure 2:**
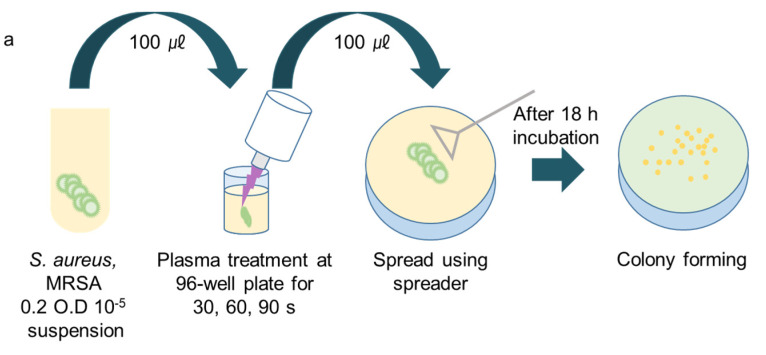

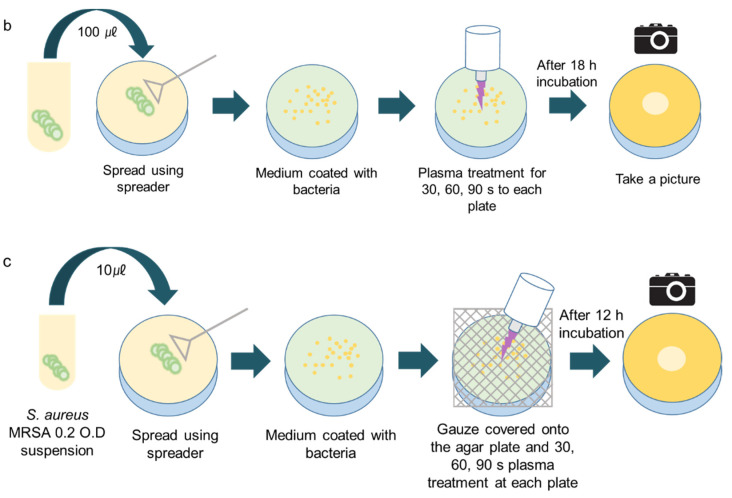
Schematic of CAPMP treatment of bacteria (**a**) in the suspension, (**b**) spread onto the agar plate, or (**c**) a dressing gauze covering the LB agar plate.

The schematics of three experimental procedures for the CAPMP treatment of bacteria are shown in Figure 2. For the treatment of the bacteria in the suspension, 100 ㎕ of the microorganism suspension at 0.2 OD was diluted to 10^−5^ and injected into a 96-well plate (cell culture plate, 96-well, SPL, Pocheon-si, Gyeonggi-do, Korea). Then, CAP was applied to the suspension for 30, 60, or 90 s by controlling Ar gas flow to prevent the microbial diluted suspension from overflowing (Figure 2a). For the treatment of the bacteria spread onto the agar plate, *S. aureus* and MRSA (100 ㎕, 0.6 OD and 10 ㎕, 0.2 OD) were spread on the LB agar plate (Ø100 and Ø60 mm, Petri Dish, SPL, Pocheon-si, Gyeonggi-do, Korea) without and with being covered by a dressing gauze, respectively (Figure 2b,c). After the tip-target distance was vertically fixed at 10 mm, CAP was applied without moving for 30, 60, or 90 s. The agar plate was incubated at 37 °C for 18 h, and the bacteria on the agar surface was analyzed by using ImageJ software (National Institutes of Health, Bethesda, MD, USA). This distance between pipette tip and gauze was fixed at 3 mm, while the gap between tip and agar plate was 10 mm. The control groups for all cases were treated with Ar gas for 90 s without plasma discharge.

The clear zone of bacteria on the agar plate was measured by ImageJ software, and the results were represented by the average and standard deviations from three independent experiments. The Student’s *t*-test analysis was performed using the Microsoft Excel program (Microsoft, Redmond, WA, USA) to quantify the difference between the experimental groups.

## 3. Results

### 3.1. CAPMP Treatment on Bacteria in the Suspension

**Figure 3:**
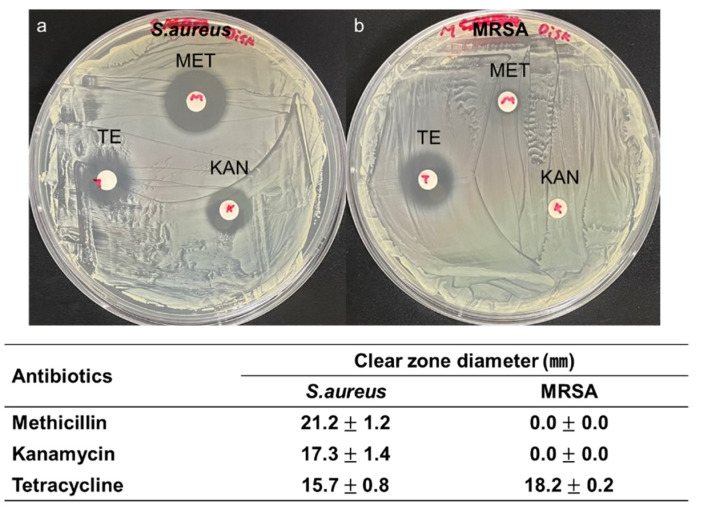
Antibiotics disk diffusion test of (**a**) *S. aureus* and (**b**) MRSA strains on the LB agar plate with a diameter of 100 mm. The diameter of the clear zone was represented by the average and standard deviation (*n* = 3).

The antibiotic resistance characteristics of MRSA were confirmed using the antibiotic disk diffusion test. Two microorganisms (*S. aureus* and MRSA) diluted to the same concentration were spread on an agar plate with a diameter of 100 mm. Three antibiotics, kanamycin (KAN 30 μg/disk, Sigma-Aldrich, St. Louis, MO, USA), tetracycline (TE 30 μg/disk, Sigma-Aldrich, St. Louis, MO, USA), and methicillin (MET 5 μg/disk, Sigma-Aldrich, St. Louis, MO, USA) were inoculated. KAN 30 μg/disk, TE 30 μg/disk, and MET were inoculated into the strains spread on the plate. Figure 3 shows the agar plate with three antibiotics after incubation at 37 °C for 18 h. The average and standard deviation (*n* = 3) of clear zone diameter in *S. aureus* plate was 17.3 ± 1.4 mm for KAN, 15.7 ± 0.8 mm for TE, and 21.2 ± 1.2 mm for MET. The value (*n* = 3) in the MRSA plate was 18.2 ± 0.2 mm for TE. However, no clear zone for MET and KAN was observed, which meant that MRSA exhibited antibiotic resistance against MET and KAN.

**Figure 4:**
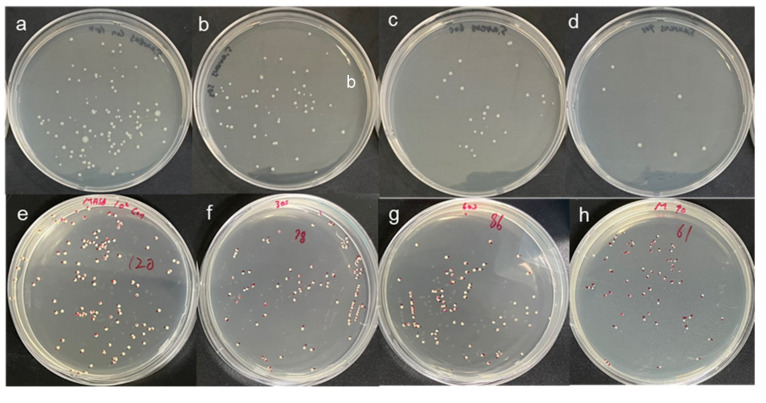

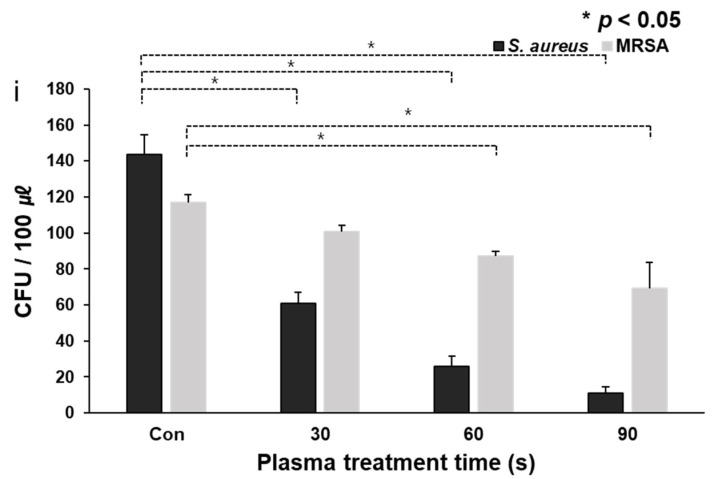
Colonies of (**a**–**d**) *S. aureus* and (**e**–**h**) MRSA on the LB agar plate with a diameter of 100 mm after CAPMP treatment on *S. aureus* or MRSA suspension. (**a**,**e**) Control applied with solely Ar gas for 90 s or (**b**–**d**,**f**–**h**) with plasma discharge for (**b**,**f**) 30, (**c**,**g**) 60, or (**d**,**h**) 90 s; (**i**) average and standard deviation of the colonies for *S. aureus* or MRSA with respect to the CAPMP treatment time (*n* = 3), *: *p* < 0.05 by *t*-test between the groups.

After the CAPMP treatment of 1–1.5 × 10^2^ CFU/100 ㎕ of the microbial suspension in a 96-well plate, the colonies formed on the LB agar plate, according to the treatment time shown in Figure 4. The figure shows the colonies of *S. aureus* (Figure 4a–d) or MRSA (Figure 4e–h) suspensions, according to the CAP application time of 30 (Figure 4b,f), 60 (Figure 4c,g), or 90 s (Figure 4d,h). The control group for *S. aureus* (Figure 4a) and MRSA (Figure 4e) suspensions were treated solely with Ar gas for 90 s. The average and standard deviation of the colonies was 124 ± 23 CFU/100 ㎕ for the untreated *S. aureus* suspension (Figure 4a). The values were 51 ± 12, 21 ± 7 and 9 ± 3 CFU/100 ㎕ when *S. aureus* in the suspension was treated for 30, 60, and 90 s, respectively.

The average and standard deviation of the untreated MRSA were 117 ± 4 CFU/100 ㎕. However, the values of the treated MRSA were 100 ± 4, 87 ± 3, and 70 ± 14 CFU/100 ㎕ when the treatment times were 30, 60, and 90 s, respectively. The developed CAPMP exhibited a feasibility for disinfection of the bacterial suspension. Compared with the control group treated solely with Ar gas for 90 s, the CAP-treated groups exhibited a considerably lower CFU, depending on the treatment time. The decrease in the CFU value corresponding to the CAP treatment was larger for *S. aureus* than for MRSA.

### 3.2. CAPMP Treatment on Bacteria Spread onto Agar Plate

**Figure 5:**
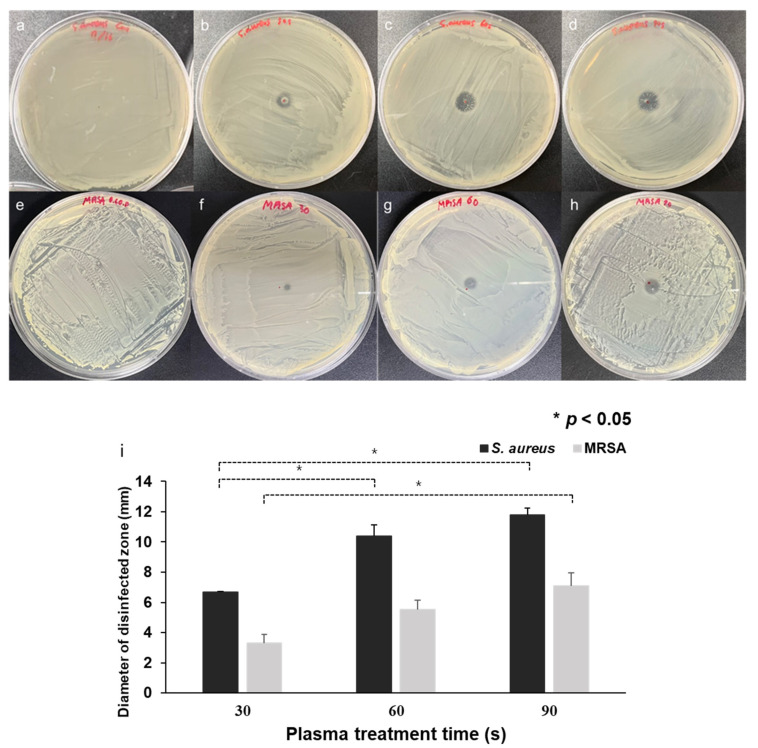
Clear zone test, according to the treatment time of CAPMP on (**a**–**d**) *S. aureus* and (**e**–**h**) MRSA spread on the agar plate with a diameter of 100 mm. (**a**,**e**) Control applied with Ar gas only for 90 s or (**b**–**d**,**f**–**h**) with plasma discharge for (**b**,**f**) 30, (**c**,**g**) 60, or (**d**,**h**) 90 s, (**i**) average and standard deviation of the clear zone diameter for *S. aureus* or MRSA with respect to the CAPMP treatment time (*n* = 3), *: *p* < 0.05 by *t*-test between the groups.

The disinfection ability of CAPMP on the bacteria spread on the agar plate with respect to the treatment time is shown in Figure 5. The figure shows the agar plates of *S. aureus* (Figure 5a–d) and MRSA (Figure 5e–h) treated with no plasma (Figure 5a,e) or with Ar plasma discharge for 30 s (Figure 5b,f), 60 s (Figure 5c,g), and 90 s (Figure 5d,h). The plate surface of the control group treated solely with Ar gas for 90 s was similar to that of the microbial culture plate without Ar gas application. This means that the growth of bacteria was not hindered by the application of Ar gas. However, the clear zone at the center of the agar plate where the CAPMP was applied was evident, and its size was dependent on the treatment time. The average and standard deviation of the clear zone diameter in the *S. aureus* plates (*n* = 3) were 6.69 ± 0.03 mm, 10.38 ± 0.73 mm, and 11.77 ± 0.45 mm when the CAPMP treatment times were 30, 60, and 90 s, respectively. The increase in the clear zone diameter in *S. aureus* plates was 55% when the CAPMP treatment time increased from 30 to 60 s, or 76% when the treatment time increased from 30 to 90 s. The value in the MRSA plates (*n* = 3) were 3.3 ± 0.56 mm, 5.51 ± 0.63 mm, and 7.1 ± 0.85 mm when the CAPMP treatment times was 30, 60, and 90 s, respectively. The increase in the clear zone diameter in MRSA plates was 67% when the CAPMP treatment time increased from 30 to 60 s, or 115% when the treatment time increased from 30 to 90 s. These results demonstrate that the CAPMP can be feasibly applied for the disinfection of the bacteria spread on the solid agar plate.

### 3.3. CAPMP Treatment on Bacteria Covered by Dressing Gauze

**Figure 6:**
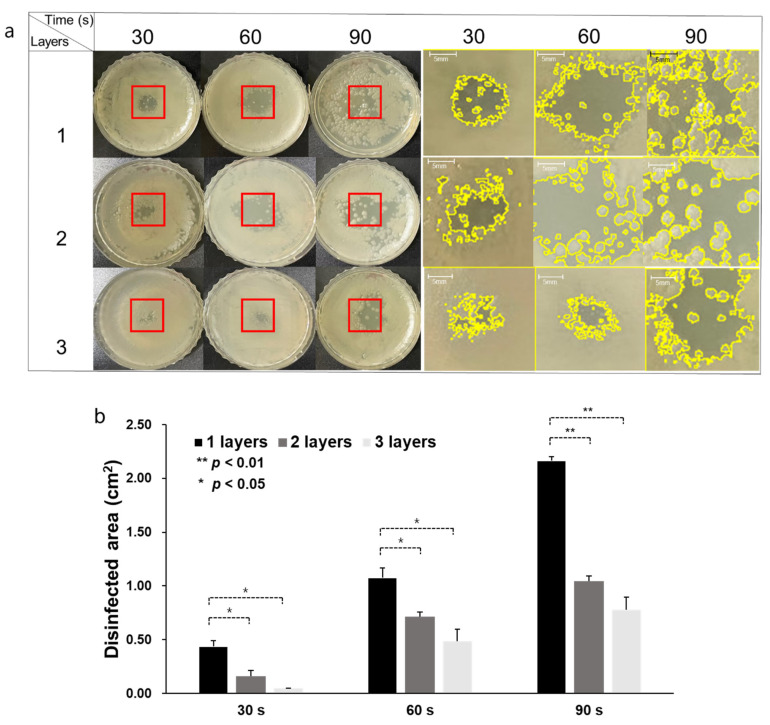
CAPMP treatment on *S. aureus* spread on the agar plate covered by single, double, or triple gauze layers. (**a**) Disinfected clear zone on the *S. aureus* agar plate, indicated by the zoomed-in red ROI shown on the right columns, with yellow lines indicating the zone boundary. (**b**) Average and standard deviation of the disinfected area analyzed using ImageJ, according to the number of gauze layers and CAPMP treatment time (*n* = 3). *: *p* < 0.05, **: *p* < 0.01 by *t*-test between the groups.

**Figure 7:**
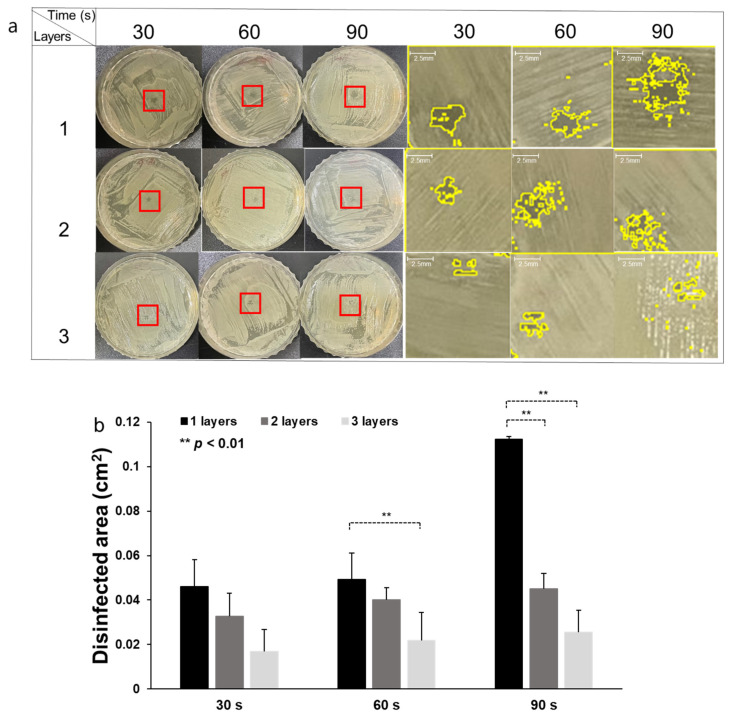
CAPMP treatment on MRSA spread on the agar plate covered by single, double, or triple gauze layers. (**a**) Disinfected clear zone on the *S. aureus* agar plate, indicated by the zoomed-in red ROI shown on the right columns, with yellow lines indicating the zone boundary. (**b**) Average and standard deviation of the disinfected area analyzed using ImageJ, according to the number of gauze layers and CAPMP treatment time (*n* = 3). *: *p* < 0.05, **: *p* < 0.01 by *t*-test between the groups.

The disinfection effect of CAPMP treatment on *S. aureus* and MRSA covered by a dressing gauze was dependent on the number of gauze layers, as shown in Figure 6 and Figure 7. Figure 6a and Figure 7a show the *S. aureus* and MRSA spread on an LB agar plate, after treatment, inoculated with bacteria, according to the number of gauze layers and treatment time. The zoomed-in red region of interest (ROI) is shown in the right columns, in which the boundary of the clear (disinfected) zone is represented by a yellow line. For the CAPMP treatment of the *S. aureus* covered by one gauze layer, the average and standard deviation (*n* = 3) of the disinfected zone area analyzed using ImageJ were 0.44 ± 0.05, 1.07 ± 0.09, and 2.16 ± 0.03 cm^2^ when the treatment times were 30, 60, and 90 s, respectively (Figure 6b). Compared with the disinfected zone area for the treatment time of 30 s, those for 60 and 90 s were 143% and 391% higher, respectively. For the treatment of the bacteria for two gauze layers, the average and standard deviation (*n* = 3) of the disinfected zone area were 0.16 ± 0.04, 0.71 ± 0.04, and 1.04 ± 0.04 cm^2^ when the treatment times were 30, 60, and 90 s, respectively. The disinfected zone area for the treatment times of 60 and 90 s was 344% and 550% higher, respectively, than that for 30 s. The values (*n* = 3) of the disinfected zone area for the three gauze layers were 0.04 ± 0.02, 0.48 ± 0.11, and 0.78 ± 0.11 cm^2^ when the treatment times were 30, 60, and 90 s, respectively. The disinfected zone area for the treatment times of 60 and 90 s was 1100% and 1850% higher, respectively, than that for 30 s. The experimental results demonstrated that the number of gauze layers affected the degree of disinfection of *S. aureus*.

For the CAPMP treatment of the MRSA for 30 s, the average and standard deviation (*n* = 3) of the disinfected area were 0.05 ± 0.01, 0.03 ± 0.01, and 0.017 ± 0.001 cm^2^ for single, double, and triple gauze layers, respectively (Figure 7b). The disinfected area for MRSA was 35% and 63% lower for two and three gauze layers, respectively, then that for one layer. When the CAPMP treatment time was 60 s, the values (*n* = 3) were 0.05 ± 0.01, 0.04 ± 0.005, and 0.02 ± 0.006 cm^2^ for single, double, and triple gauze layers, respectively. The disinfected area for two and three gauze layers was 18% and 59% lower, respectively, then that for one layer. Finally, the values (*n* = 3) for the treatment time of 90 s were 0.11 ± 0.01, 0.05 ± 0.01, and 0.03 ± 0.01 cm^2^ for single, double, and triple gauze layers, respectively. The disinfected area for one gauze layer was the 60% and 78% higher than that for two or three gauze layers, respectively. Therefore, the disinfection effect of CAPMP on MRSA was diminished, since the arrival of the plasma on the bacteria was hindered by the number of gauze layers covering the agar plate. However, the increase in the treatment time led to an increase in the disinfected area of the agar plate covered with gauze.

## 4. Discussion

In this study, the feasibilities of a CAPMP for the disinfection of *S. aureus* and MRSA in culture media, agar plates, and agar plates covered with dressing gauze were evaluated. The experimental protocols were successfully designed to develop the effective CAPMP on the disinfection of bacteria. The temperature of plasma plume is considered as an important factor that can affect to the survival of cells and microorganisms. Hence, the temperature of plasma plume at a 10 mm distance from the pipette tip was fixed at 45 °C. The similar viability of bacteria was determined after keeping the cells at 45 °C for 5 min, indicating that the temperature of the plasma plume showed a negligible disinfection effect to bacteria [42,43]. Therefore, it is worthy to note that the effect of the plasma plume temperature on the plasma disinfection to bacteria could be ignored.

A decrease in the viability of *S. aureus* and MRSA in the culture media was observed with increasing CAPMP treatment time (Figure 4). Furthermore, the area of the disinfected zone (clear zone) of the bacteria on the agar plate was increased with the increase of the CAPMP treatment time. The area of the disinfected zone was not significantly increased for *S. aureus* when the CAPMP treatment time was increased by more than 90 s (not shown). Additionally, the disinfection effect of CAPMP on MRSA was less effective than that on *S. aureus*, due to the relative resistance of MRSA to plasma, as compared to *S. aureus* [44,45].

For the disinfection effect of plasma through medical gauze, the plasma could pass through easily and reach the infected area, owing to the support of the small holes in a grid pattern, in the gas exchange. Thus, the plasma easily passed through the gauze and exhibited the disinfection effect to bacteria on the agar medium. Due to the increase of gauze’s thickness, the denser lattice pattern prevented the plasma absorption, leading to less generation of radicals or reactive species, resulting in a decrease of the disinfected effect (Figure 6 and Figure 7). Under treatment with gauze, the plasma plume split while passing through the grid pattern of medical gauze, leading to the diffusion of plasma, thus making the irregular disinfection area [46]. Consequentially, the plasma area of the disinfected zone could be expanded up to about 2 cm^2^ by the direct and indirect effect of the plasma [47]. It is well known that the radicals, reactive species, and UV radiation can affect the microbial cell membrane or organelles to kill the cells via the leakage of molecules into cells, as well as DNA or protein modification [48,49]. Due to the reduction of the bactericidal effect, when exposed to increased layers of medical gauze or other obstacles, the practical application of the developed CAPMP will be evaluated in animal models with plasma treatment time, plasma frequency, and scanning method.

## 5. Conclusions

This study developed a CAPMP that outputs an Ar plasma plume through a tube with an inner radius of 180 μm for disinfection in a tiny area. The developed CAPMP can be feasibly applied for the disinfection of *S. aureus* and MRSA diluted in liquid media, spread on a solid agar plate, and covered by dressing gauzes. The experimental results demonstrated that the disinfection was influenced by the CAPMP treatment time and number of gauze layers. The increase in the treatment time on the bacterial suspension led to a decrease in the number of colonies. Furthermore, the disinfected zone area in the bacteria spread on the agar plate increased with the treatment time. The degree of disinfection decreased with an increase in the number of gauze layers, hindering the arrival of plasma onto the bacteria.
